# Supplementary material for: Observation of dichotomic field-tunable electronic structure in twisted monolayer-bilayer graphene
Source: Nat Commun. 2024 May 3;15:3737. doi: 10.1038/s41467-024-48166-8 (PMC11068895; doi:10.1038/s41467-024-48166-8)
Supplement: Supplementary file 1 — Supplementary Information [file 41467_2024_48166_MOESM1_ESM.pdf]

Supplementary Information

Hongyun Zhang[1,†], Qian Li[1,†], Youngju Park[2], Yujin Jia[3,4], Wanying Chen[1], Jiaheng Li[3,4], Qinxin Liu[1], Changhua Bao[1], Nicolas Leconte[2], Shaohua Zhou[1], Yuan Wang[1], Kenji Watanabe[5], Takashi Taniguchi[6], Jose Avila[7], Pavel Dudin[7], Pu Yu[1,8], Hongming Weng[3,4,9], Wenhui Duan[1,8,10], Quansheng Wu[3,4], Jeil Jung[2,11] & Shuyun Zhou[1,8,*]

[1]*State Key Laboratory of Low-Dimensional Quantum Physics and Department of Physics, Tsinghua University, Beijing 100084, P. R. China*

[2]*Department of Physics, University of Seoul, Seoul 02504, Korea*

[3]*Beijing National Laboratory for Condensed Matter Physics and Institute of Physics, Chinese Academy of Sciences, Beijing 100190, P. R. China*

[4]*University of Chinese Academy of Sciences, Beijing 100049, P. R. China*

[5]*Research Center for Electronic and Optical Materials, National Institute for Materials Science, 1-1 Namiki, Tsukuba 305-0044, Japan*

[6]*Research Center for Materials Nanoarchitectonics, National Institute for Materials Science, 1-1 Namiki, Tsukuba 305-0044, Japan*

[7]*Synchrotron SOLEIL, L' Orme des Merisiers, Departamentale 128, 91190 Saint-Aubin, France*

[8]*Frontier Science Center for Quantum Information, Beijing 100084, P. R. China*

[9]*Songshan Lake Materials Laboratory, Dongguan, Guangdong 523808, P. R. China*

[10]*Institute for Advanced Study, Tsinghua University, Beijing 100084, P. R. China*

[11]*Department of Smart Cities, University of Seoul, Seoul 02504, Korea*

† *These authors contributed equally to this work.*

Correspondence should be sent to syzhou@mail.tsinghua.edu.cn.

Contents:

Supplementary Note 1. Sample preparation.

Supplementary Note 2. Flat bands observed in tMBG with twist angles of 2.2°, 2.6° and 3.0°.

Supplementary Note 3. Doping effect and selective enhancement of 2 ML and 1 ML bands at different gating voltages.

Supplementary Note 4. Switching between electron and hole bands by reversing the bias voltage.

Supplementary Note 5. Selectively enhanced contribution from different stacked graphene layers under bias voltage.

Supplementary Note 6. Theoretical calculations.

Supplementary Note 7. ARPES measurement geometry.

Supplementary information

1 Sample preparation.

The tMBG sample was prepared by using the clean dry transfer method[1,2] as schematically shown in Supplementary Figure 1. First, the graphene flake with both monolayer and bilayer parts connected together was exfoliated onto a clean SiO_2/Si substrate (Supplementary Figure 1a). A thin BN flake attached to PVA/PDMS (Polyvinyl Alcohol/Polydimethylsiloxane) was then positioned above the graphene under an optical microscope to pick up the bilayer graphene part (Supplementary Figure 1b). The monolayer graphene on the SiO_2/Si substrate was rotated by the desired angle and picked up by bilayer-graphene/BN/PVA/PDMS to form the tMBG/BN/PVA/PDMS structure (Supplementary Figure 1c and the optical image in Supplementary Figure 2b). Subsequently, the tMBG/BN/PVA/PDMS was flipped over, and the tMBG/BN/PVA was picked up by another PDMS stamp to form PVA/BN/tMBG/PDMS structure (Supplementary Figure 1d). The PVA film was dissolved by immersing the entire structure in water for several hours, and the tMBG/BN was transferred onto a graphite flake (Supplementary Figure 1e), which was in contact with the gold-plated pattern as the bottom gate electrode. Finally, two narrow pieces of graphite were used to electrically connect tMBG and the gold-coated pattern (Supplementary Figure 1f and the optical image in Supplementary Figure 2c) to ensure good electrical conductivity for ARPES measurements. The gold-plated pattern was connected to the gating electrode of the sample holder by wire bonding.

The twist angle can be determined by combining NanoARPES and lateral force AFM (L-AFM) measurements. For the L-AFM measurements, silicon nitride probes are used to obtain the lateral force and topography image under the contact mode. We note that L-AFM measurement is particularly sensitive to the moiré superlattice period because the stick-slip effect would occur at the moiré superlattice scale [3]. After extracting the moiré superlattice period λ_m from L-AFM measurements, the twist angle θ can be further determined by $\lambda_m = a/(2sin(\theta/2))$.

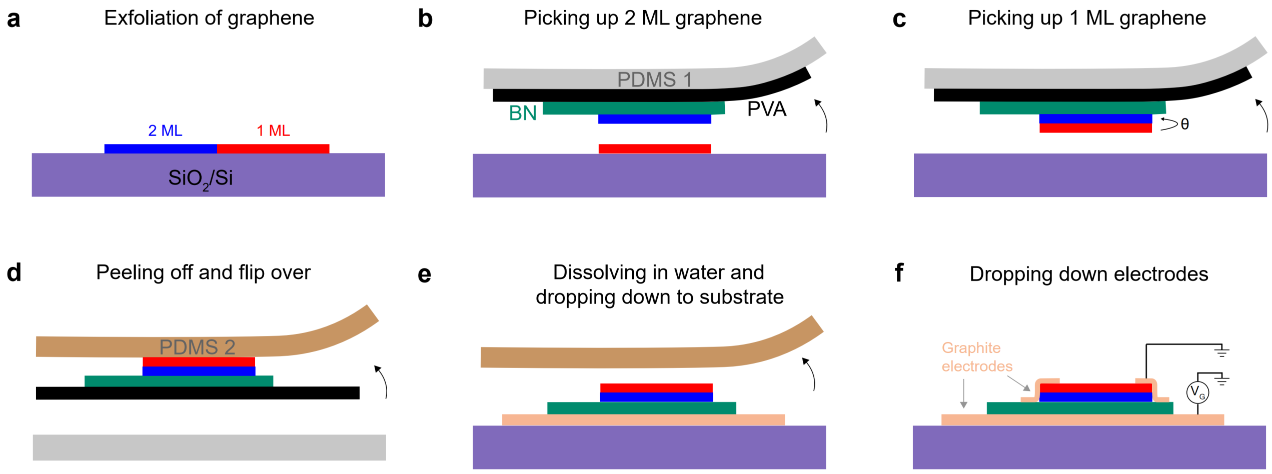

Supplementary Figure 1. Preparation of the gate-tunable tMBG samples. a-f, Schematic drawings of the sample preparation process including the exfoliation of graphene, picking up 2 ML and 1 ML graphene with a rotation angle, peeling off and flipping over, dissolving of PVA in water and dropping down the sample and graphite electrodes to the substrate.

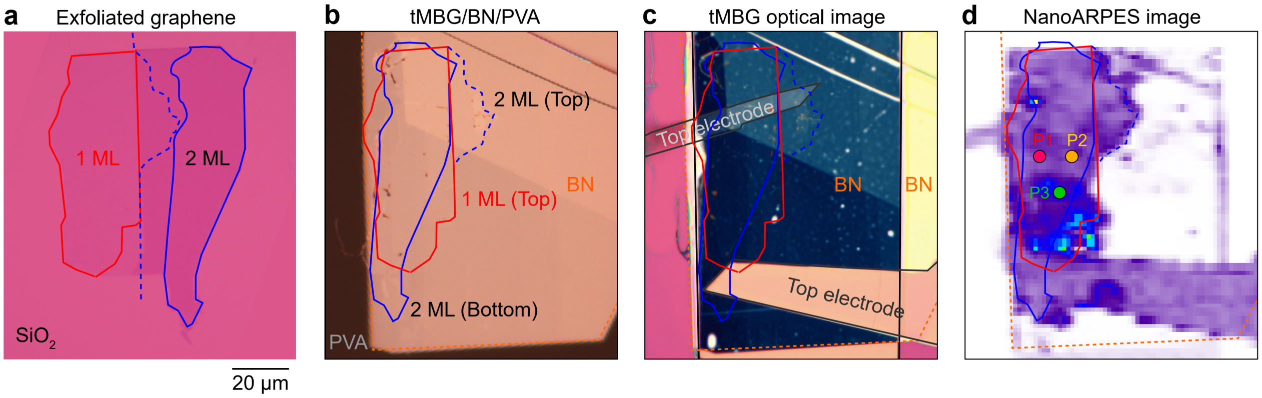

Supplementary Figure 2. Optical images and NanoARPES spatial intensity map of the tMBG sample. **a**, The optical image of the exfoliated graphene on SiO_2/Si substrate, from which the regions of 1 ML (red curve) and 2 ML (blue curve) graphene are identified. The blue dashed curve indicates the break line when picking up the graphene. **b**, An optical image during the sample preparation process that corresponds to the Supplementary Figure 1c. **c,d**, Optical image of the tMBG sample and the spatial intensity map obtained by integrating the measured intensity from -1 eV to E_F. The colored spots P_1, P_2 and P_3 indicate the measurement positions with twist angles of 2.2°, 2.6° and 3.0°, respectively.

2 Flat bands observed in tMBG with twist angles of 2.2°, 2.6° and 3.0°.

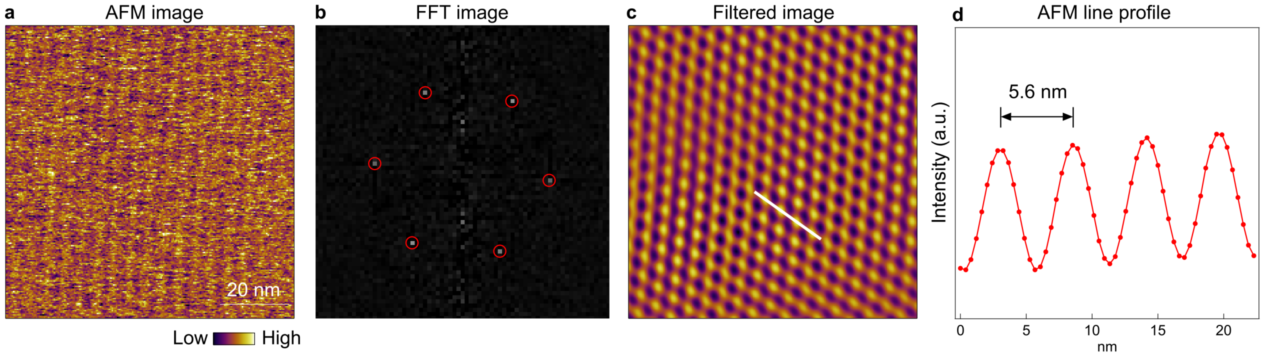

Supplementary Figure 3. Moiré period revealed by AFM measurements. a, AFM image measured near the center position that corresponds to the region of 2.6° at P_2. The white scale bar indicates 20 nm. **b,c**, The FFT results and filtered image of **a**. **d**, Line profiles along the white line in **c**, from which the moiré period is determined to be 5.6 $\pm$ 0.2 nm. The measured real space moiré period corresponds to the twist angle of 2.6° determined from ARPES measurements.

The tMBG sample contains regions with slightly different twist angles, and the evolution of the electronic structure can be revealed by NanoARPES measurements. The twist angle can be determined both from the real space moiré period measured by atomic force microscope (AFM) (Supplementary Figure 1) and the separation between the K points of the top 1 ML graphene (K_1) and the bottom 2 ML graphene (K_2) (Supplementary Figure 4). Supplementary Figure 4 shows electronic structures measured at 3 spots with twist angles of 2.2°, 2.6° and 3.0° marked as red, orange and green dots in Supplementary Figure 2d. The bandwidth of the flat band increases from a nearly flat band at 2.2° (Supplementary Figure 4f) to a more "M"-shaped behavior at 2.6° and 3.0° (Supplementary Figure 4m,t) with increasing twist angle. Meanwhile, the gap (pointed by black arrows in Supplementary Figure 4g,n,u) moves toward higher binding energy. The direct

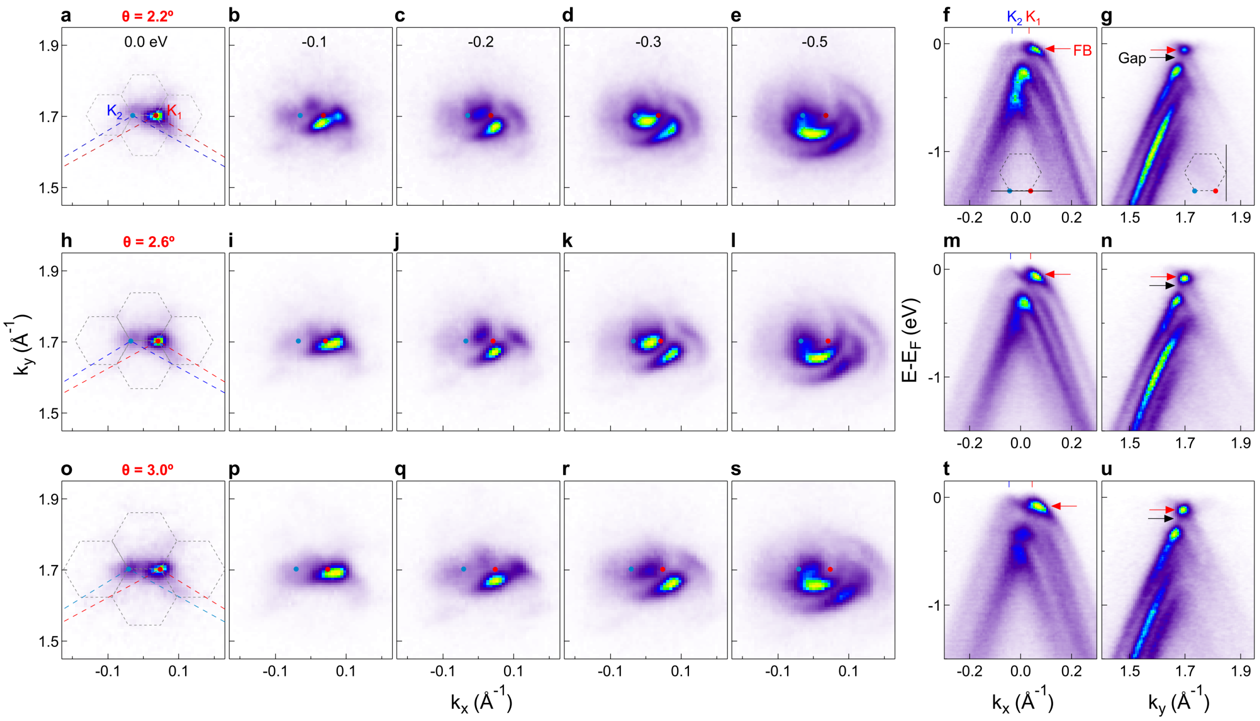

Supplementary Figure 4. Flat band observed in 2.2°, 2.6° and 3.0° tMBGs. a-e, ARPES intensity maps of 2.2° tMBG at different energies, where the dispersion from the top 1 ML graphene and bottom 2 ML graphene and the replica bands are clearly resolved. **f,g**, Dispersion images of 2.2° tMBG cutting through high-symmetry directions as indicated by the insets. **h-l**, ARPES intensity maps of 2.6° tMBG at different energies. **m,n**, Dispersion images of 2.6° tMBG with measured directions similar with **f,g**. **o-s**, ARPES intensity maps of 3.0° tMBG at different energies. **t,u**, Dispersion images of 3.0° tMBG with measured directions similar with **f,g**.

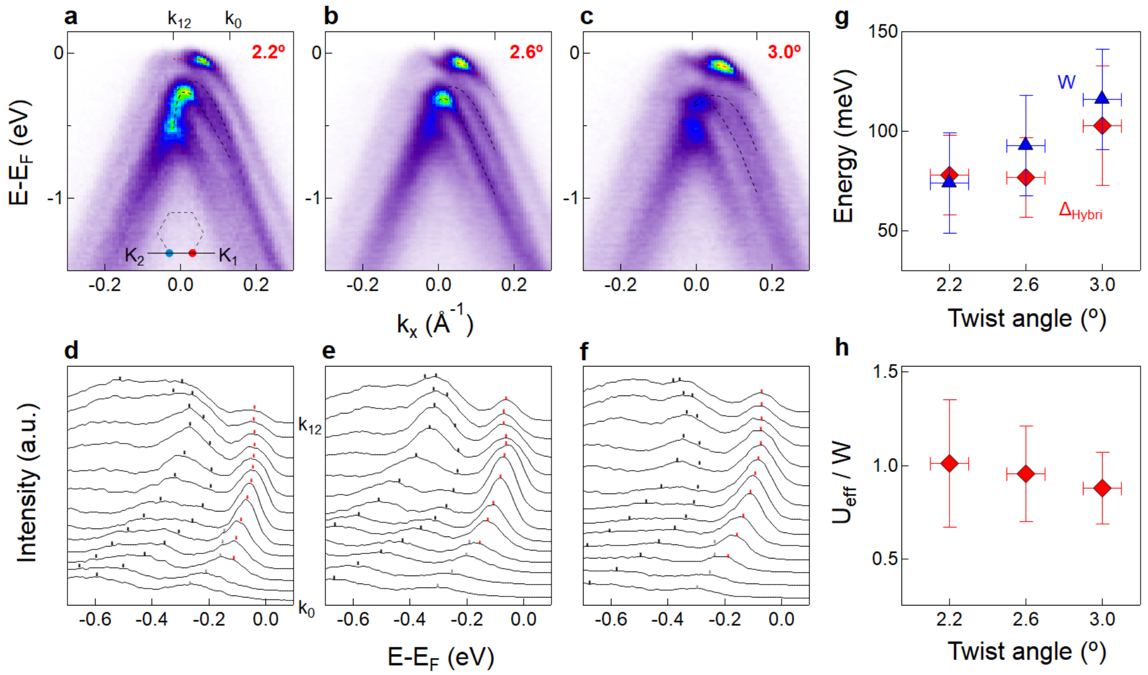

Supplementary Figure 5. Twist angle dependence of the flat band and hybridization gap by cutting through both K_1 and K_2 points. a-c, Dispersion images of 2.2°, 2.6° and 3.0° tMBG by cutting through K_1 and K_2 points. **d-f**, Energy distribution curves (EDCs) to extract the bandwidth of the flat band and the hybridization gap. **g**, Twist angle dependence of the extracted bandwidth of the flat band (w) and the hybridization gap (Δ_{Hybri}). **h**, Evolution of the effective correlation strength U_{eff}/W at different twist angles. Data in **g,h** are presented as extracting values with different twist angles from **d-f** , with error bars representing the standard error.

observation of the evolution of flat band with twist angle agrees with the band hybridization picture schematically illustrated in Supplementary Figure 1c.

The high-quality ARPES data allow to extract the bandwidth of the flat band and the hybridization gap size as shown in Supplementary Figure 5. By extracting fine dispersion of the flat band cutting both K_1 and K_2 points, we can obtain the effective correlation strength U_{eff}/W at different twist angles, which shows that 2.2° falls into the correlated regime with $U_{eff}/W > 1$ (Supplementary Figure 5h).

3 Doping effect and selective enhancement of 2 ML and 1 ML bands at different gating voltages.

The application of a bias voltages not only tunes the doping of tMBG, but also selectively enhances the 2 ML and 1 ML bands due to the existence of electric field. Supplementary Figure 6a-g shows the dispersion images measured as indicated by the inset, from which the hole doping to electron doping effect is observed from negative to positive bias voltages as schematically drawn in Supplementary Figure 6h-n. From 0 V to -20 V, the flat band feature (indicated by blue arrows) moves up in energy due to the hole doping at negative bias voltage. From 0 V to 30 V, the electron doping shifts the bands down in energy of 140 $\pm$ 30 meV. The evolution of flat band and characteristic features can be better resolved in the schematic drawing in Supplementary Figure 6h-n. The carrier density n can be calculated from the geometric capacitance of the tMBG device. The tMBG gating device is viewed as a parallel plate capacitor with carrier density[4,5] of $n = \epsilon_0 \epsilon_r V_g/ed$, where ϵ_0 = 1 and ϵ_r = 4 are the permittivity of the vacuum and BN substrate, d = 47 nm is the thickness of the BN determined by AFM, e is the elementary charge, and V_g is the applied bias voltage. Based on this, the carrier density at V_g = 30 V is calculated to be 1.4 $\times$ 10^{13} /cm^2, which is in agreement with the energy shift (ΔE) energy shift of 140 meV by $\Delta E^2 = \pi n \hbar^2 v_F^2/2$.

The bias voltage also leads to dichotomic field-tunable flat band dispersion versus flatter or dispersive as shown in Supplementary Figure 7. First of all, from theoretical calculations, it is clear that for Δ = -100 meV, the band near E$_F$ from 1 ML graphene (guided by red dashed line in Supplementary Figure 7a) is flatter than that for Δ = 100 meV (guided by black dashed

line in Supplementary Figure 7e). Secondly, when overplotting theoretical calculations with our experimental data (Supplementary Figure 7f-j), there is an overall agreement between experimental results and theoretical calculations. The good agreement between the ARPES data and the overplotted calculations (Supplementary Figure 7f-j) confirms the dichotomic field-tunable flat band under gating. Moreover, the bias voltage leads to carrier redistribution among different graphene layers, which can be reflected by the dichotomic enhancement of 2 ML and 1 ML bands under opposite bias voltages. As shown in Supplementary Figure 8, while the 2 ML bands become stronger (blue arrow) and comparable to the 1 ML bands (red arrow) at -20 V (Supplementary Figure 8a,f), it become almost undetectable at 20 V (Supplementary Figure 8e,j).

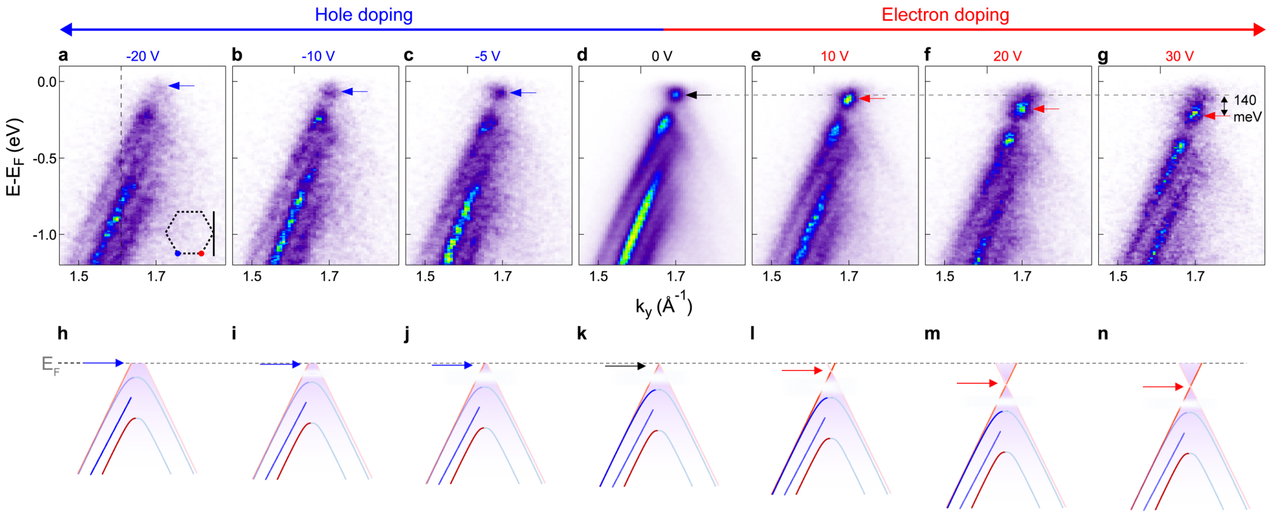

Supplementary Figure 6. Doping effect by applying bias voltages. a-g, Dispersion images measured along the black line as shown in the inset. **h-n**, Schematic drawing of dispersion images in **a-g** to show the hole and electron doping effect under negative and positive gating voltages respectively.

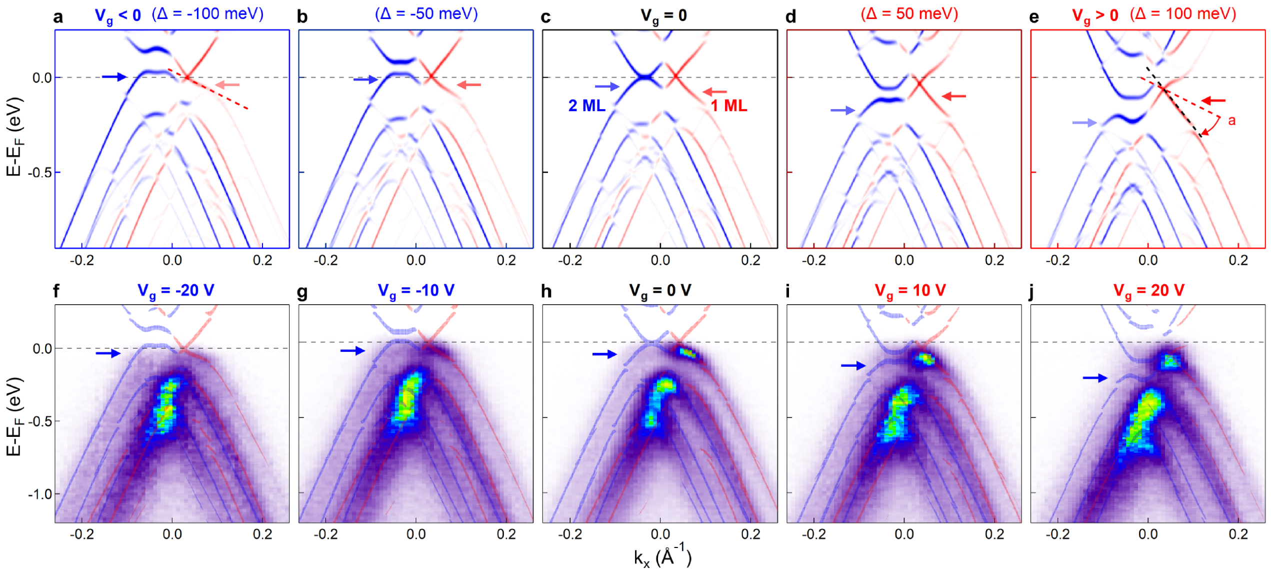

Supplementary Figure 7. Flatter versus dispersive flat band under negative or positive gating voltages. a-e, Calculated spectrum under interlayer potential of -100, -50, 0, 50 and 100 meV. **f-j**, High-quality ARPES measured dispersion images along K_1-K_2 direction under bias voltages of -20, -10, 0, 10 and 20 V. Calculation results are over-plotted onto **f-j** to have a better comparison, which shows good agreement between experiments and calculations.

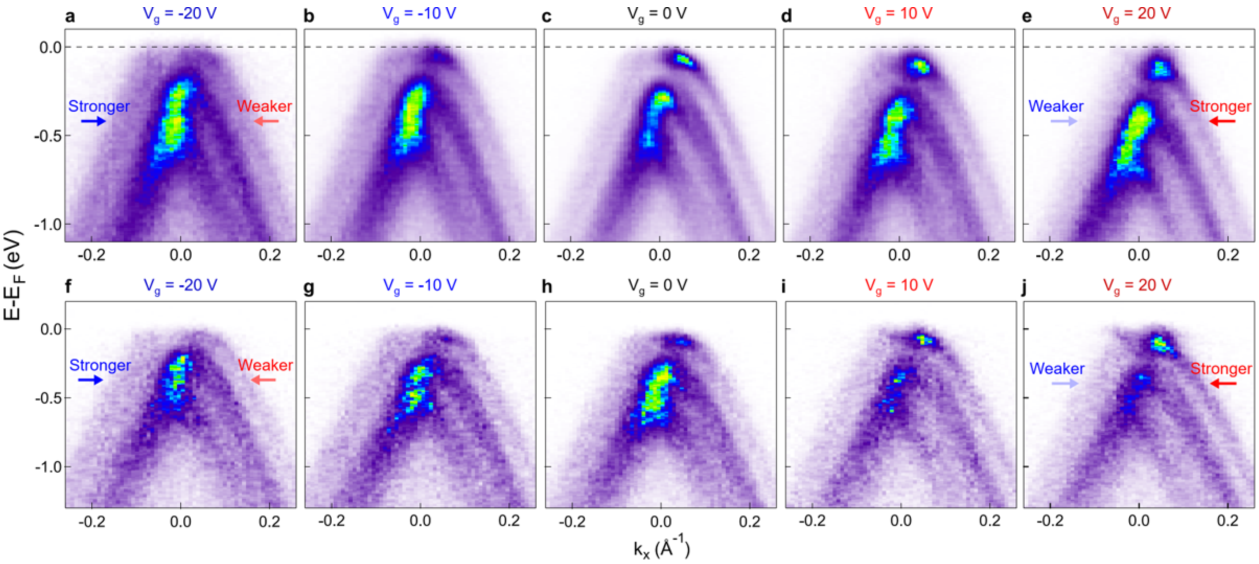

Supplementary Figure 8. Two sets of experimental data with different statistics to show the relative spectral weight transfer upon gating. a-e, ARPES measured dispersion images with higher statistics along K_t-K_b direction upon changing gating from -20 V to 20 V. **f-j**, ARPES measured dispersion images with lower statistics along K_t-K_b direction upon changing gating from -20 V to 20 V.

4 Switching between valence and conduction bands by reversing the bias voltage.

The tMBG bands show interesting similarity between valence bands at positive bias voltage and conduction bands at negative bias voltage (by comparising Supplementary Figure 9c,d), suggesting a switching between the electron and hole bands by reversing the bias voltage.

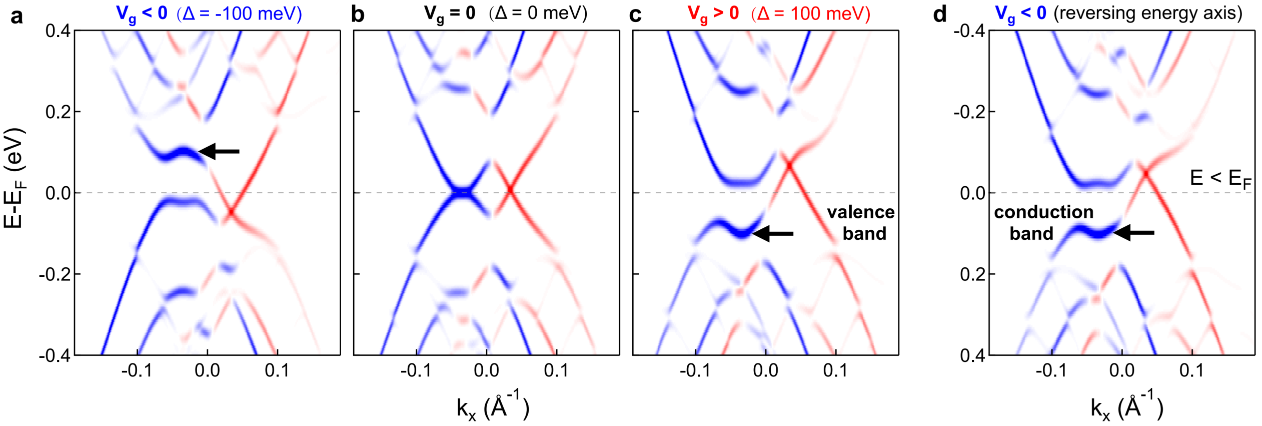

Supplementary Figure 9. Switching between electron and hole bands by reversing the bias voltage. **a-c**, Calculated spectra under the bias voltage of $V_g < 0$ (Δ = -100 meV), $V_g = 0$ and $V_g > 0$ (Δ = 100 meV), respectively. Red and blue colors representing the contribution from 1 ML and 2 ML graphene. **d**, Same calculated spectrum as **a**, except that the energy axis is reversed for easy comparison with **c**. Comparison between **c** with **d** suggests an overall similar behavior of the electron and hole bands under opposite bias voltages.

5 Selectively enhanced contribution from different stacked graphene layers under bias voltage.

The calculated layer-resolved energy contours in Fig. 4 show selectively enhanced spectral weight and pocket size under positive and negative bias voltage. Supplementary Figure 10 shows the

comparison of calculated DOS at -0.2 eV for tBLG, tMBG and tDBG structures. The selectively enhanced contribution from top layers (Supplementary Figure 10d-f) under positive bias voltage leads to similarity between tBLG and tMBG structures. While under negative bias voltage, the much weaker contribution from the extra top layer in tDBG (Supplementary Figure 10l) can explain the similarity between the energy contours for tMBG and tDBG (Supplementary Figure 10h,i).

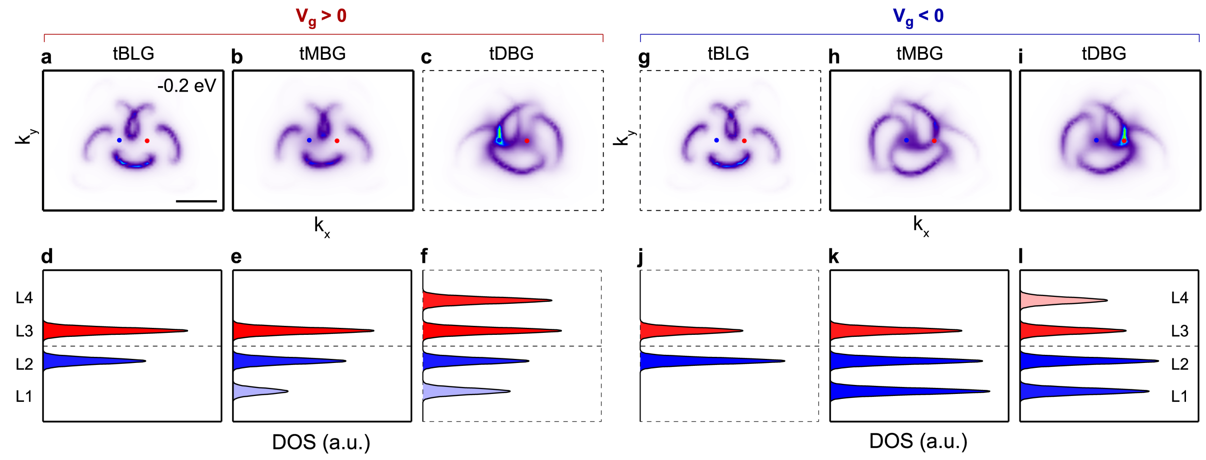

Supplementary Figure 10. Selectively enhanced contribution from different stacked graphene layers under bias voltage. a-c, Calculated energy contours at -0.2 eV for tBLG, tMBG and tDBG under positive bias voltages with interlayer potential difference of Δ = 100 meV. The scale bar in **a** is 0.1 Å^{-1}. **g-i**, Calculated DOS at -0.2 eV for tBLG, tMBG and tDBG under positive bias voltages with Δ = 100 meV. **g-i**, Calculated energy contours at -0.2 eV for tBLG, tMBG and tDBG under negative bias voltages with Δ = -100 meV. **j-l**, Calculated DOS at -0.2 eV for tBLG, tMBG and tDBG under positive bias voltages with Δ = -100 meV.

6 Theoretical calculations.

The electronic structure calculations are performed by using a real-space tight-binding model, where we define the Hamiltonian for tBLG, tMBG, and tDBG as

$$H = \sum_{i,j} t_{ij} c_i^\dagger c_j + \sum_i (\varepsilon_i + V_{\ell \in i}) c_i^\dagger c_i \tag{1}$$

where ε_i and t_{ij} are the on-site potential energy of atom i and the hopping parameter between atom i and j. We use the effective tight-binding model parameters for ε_i and t_{ij} using either the monolayer [6] version or the Bernal bilayer [7] version depending on which part of the system is under consideration. We set the effective nearest hopping term $t_0 = -3.1$ eV and the interlayer hopping scaling factor S = 0.895 for the Scaled Hybrid Exponential (SHE) model[8], to effectively match the Fermi velocity $v_F \approx 1 \times 10^6$ m/s and the magic angle 1.08° of tBLG. In the calculations, a commensurate superlattice is used, and here we use a twist angle of 2.28° to perform the calculations, which is the closest commensurate twist angle near 2.2°. In order to describe the electric field effect after applying bias voltages, we label the graphene layers from L_1 (bottom layer) to L_4 (top layer) as indicated by Fig. 4, and the potential energy for different layers are set to be $V_1 = -V_4 = -3\Delta/2$ and $V_2 = -V_3 = -\Delta/2$. We note that a positive bias voltage $V_g > 0$ gives rise to $\Delta > 0$, leading to an induced electric field E_{ind} pointing from monolayer to bilayer graphene, where the total electric field contains $E_{total} = E_{ext} + E_{ind}$ contains the external electric field E_{ext} and the induced electric field E_{ind}. An adjustable chemical potential μ is used to introduce an overall shift in the tBLG, tMBG and tDBG bands to allow a fair comparison between bands at specific energy cuts (Fig. 4).

We perform classical structural relaxation implemented in LAMMPS [9,10]. In graphene systems, the inter-layer interaction acts differently than intra-layer one due to the van der Waals interaction, and they are usually treated with different empirical potentials. Therefore, we use different empirical inter-atomic potentials when considering the inter-layer and intra-layer relaxation. For inter-layer interactions, we used an inter-layer potential developed for graphene and hBN systems[11,12]. For intra-layer interactions, we used the reactive empirical bond order potential [13].

To simulate the intensity of the ARPES data, we calculate the sublattice-resolved spectral functions using a band-unfolding method[14–16] based on the one-particle Green's function of the moiré supercell (SC):

$$\hat{G}(z) = \sum_{I,\boldsymbol{k}_{\mathrm{SC}}} \frac{\left|\Psi^{\mathrm{SC}}_{I\boldsymbol{k}_{\mathrm{SC}}}\right\rangle\left\langle\Psi^{\mathrm{SC}}_{I\boldsymbol{k}_{\mathrm{SC}}}\right|}{z - \varepsilon_{I\boldsymbol{k}_{\mathrm{SC}}}} \tag{2}$$

where $\varepsilon_{I\boldsymbol{k}_{\mathrm{SC}}}$ and $|\Psi^{\mathrm{SC}}_{I\boldsymbol{k}_{\mathrm{SC}}} >$ are eigenenergies and eigenvectors of the folded system specified by the band index I and the momentum vector $\boldsymbol{k}_{SC}$, the spectral function of sublattice $\alpha = A_1, B_1, A_2, ..., B_4$ can be defined as

$$\begin{aligned} A_\alpha(\boldsymbol{k}, \varepsilon) &= \left\langle \boldsymbol{k} \left| -\frac{1}{\pi} \mathrm{Im}\left[\hat{G}(\varepsilon + i\eta)\right] \right| \boldsymbol{k} \right\rangle_\alpha \\ &= \sum_{I,\boldsymbol{k}_{\mathrm{SC}}} | < \boldsymbol{k} | \Psi^{\mathrm{SC}}_{I\boldsymbol{k}_{\mathrm{SC}}} >_\alpha |^2 \\ &\quad \left(-\frac{1}{\pi} \mathrm{Im}\left[\frac{1}{\varepsilon - \varepsilon_{I\boldsymbol{k}_{\mathrm{SC}}} + i\eta}\right]\right) \end{aligned} \tag{3}$$

where $|\boldsymbol{k} >$ is the final state in the unfolded space, and the first term in the summation corresponds to the spectral weights of the state $|\Psi_{I\boldsymbol{k}_{SC}} >$. The spectral weights can be estimated using the

atomic orbital basis $|\boldsymbol{R}+\tau_\alpha>$ as

$$\begin{aligned}<\boldsymbol{k}|\Psi^{\text{SC}}_{I\boldsymbol{k}_{\text{SC}}}>_\alpha &=<\boldsymbol{k}|\left[\sum_{i\in\alpha}U^{\boldsymbol{R}_i+\tau_\alpha}_{I\boldsymbol{k}_{\text{SC}}}|\boldsymbol{R}_i+\tau_\alpha>\right] \\ &=\sum_{i\in\alpha}U^{\boldsymbol{R}_i+\tau_\alpha}_{I\boldsymbol{k}_{\text{SC}}}\,e^{-i\boldsymbol{k}\cdot(\boldsymbol{R}_i+\tau_\alpha)}.\end{aligned} \tag{4}$$

We calculate spectral weights by $|<\boldsymbol{k}|\Psi^{\text{SC}}_{I\boldsymbol{k}_{\text{SC}}}>_\alpha|^2=|\sum_{i\in\alpha}U^{\boldsymbol{R}_i+\tau_\alpha}_{I\boldsymbol{k}_{\text{SC}}}\,e^{-i\boldsymbol{k}\cdot(\boldsymbol{R}_i+\tau_\alpha)}|^2$, and selectively sum over all the states of $\varepsilon=\varepsilon_{I\boldsymbol{k}_{SC}}$ with the small Lorentzian broadening of $\eta=0.02$ eV. Projection to the atomic orbitals basis $|R_i+\tau_\alpha>$ as of the eigenvector-components $U^{\boldsymbol{R}_i+\tau_\alpha}_{I\boldsymbol{k}_{\text{SC}}}=<R_i+\tau_\alpha|\Psi^{\text{SC}}_{I\boldsymbol{k}_{\text{SC}}}>$ allows us to tell apart the spectral weights attributed from each sublattice ($\alpha=A_1,B_1,A_2,...,B_4$). This band unfolding method effectively obtains the weights for each crystal momentum vector k_{SC} from the supercell, matching the vector k from the reference unit cell through k = k_{SC} + G_{SC} (here G_{SC} is the supercell reciprocal lattice vector). We repeat this unfolding approach for each of the atomic orbitals of each atom in the moiré supercell, thus obtaining the spectral weight for each sublattice in each layer, hence allowing us to plot layer and sublattice resolved contributions. The main code we used is called the WannierTools[17], which is an open source software in the GitHub that studies the physical properties of a given tight-binding model. Supplementary Figure 11 shows the comparison between the calculated band structure before (Supplementary Figure 11a) and after band unfolding into the top graphene Brillouin zone (Supplementary Figure 11b), which shows overall agreement, meanwhile the band unfolding results (Supplementary Figure 11b) can be better compared with the experimental results.

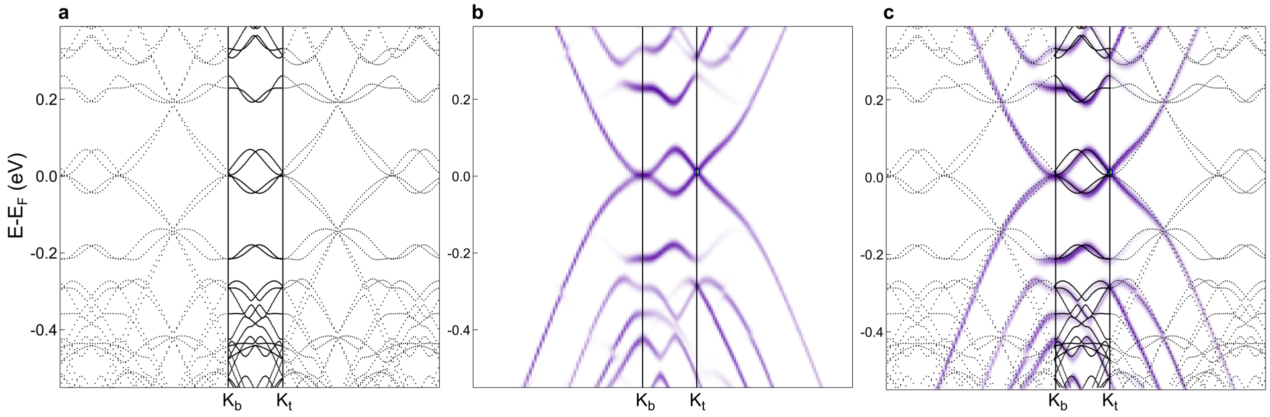

Supplementary Figure 11. Comparison between the calculations with and without band unfolding procedure. a, Calculated band structure in the moiré Brillouin zone. **b**, Calculated band structure after band unfolding to the graphene Brillouin zone. **c**, Comparison between the calculations with and without band unfolding by directly folding **a** onto **b**.

7 ARPES experimental geometry.

Supplementary Figure 12 shows the shematic drawing of ARPES measurement geometry. The light is set to p-polarized and the analyzer slit direction is horizontal. Therefore, the measurement direction is along Γ-K (here K indicates the middle point between the top (K_t) and bottom (K_b) graphene BZ corners), which is in the scattering plane (pink plane).

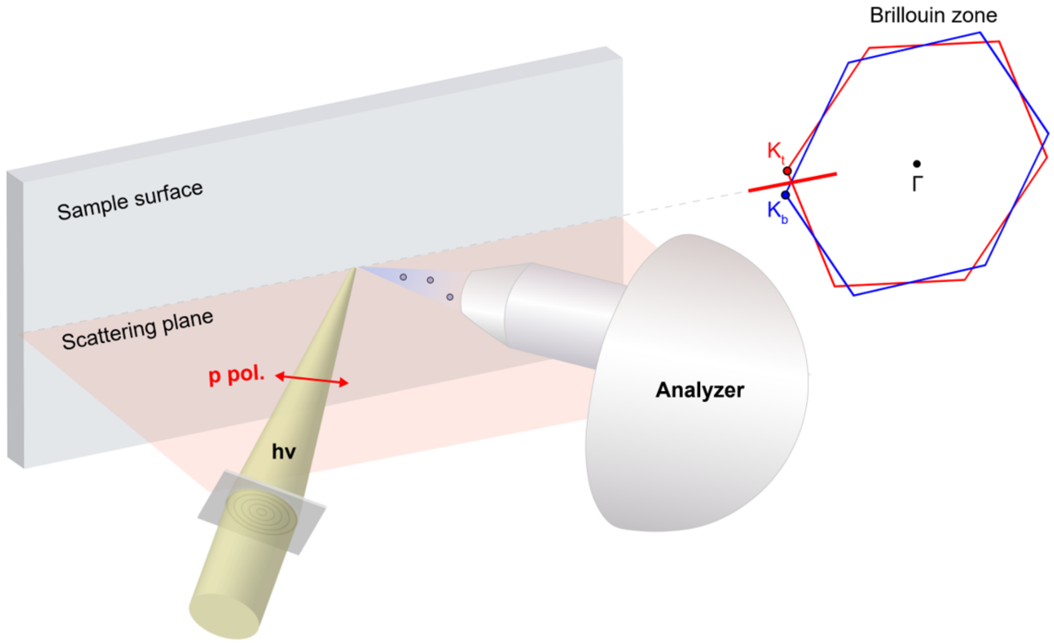

Supplementary Figure 12. ARPES measurement geometry. The light polarization is linear horizontal (p-polarized) as indicated by the red arrow. The right panel represents the Brillouin zone with the red line indicating the measurement direction.

1. Wang, L. *et al.* One-dimensional electrical contact to a two-dimensional material. *Science* **342**, 614–617 (2013).

2. Cao, Y. *et al.* Superlattice-induced insulating states and valley-protected orbits in twisted bilayer graphene. *Phys. Rev. Lett.* **117**, 116804 (2016).

3. Zhang, S. *et al.* Dual-scale stick-slip friction on Graphene/$h-$BN moiré superlattice structure. *Phys. Rev. Lett.* **128**, 226101 (2022).

4. Nguyen, P. V. *et al.* Visualizing electrostatic gating effects in two-dimensional heterostructures. *Nature* **572**, 220–223 (2019).

5. Joucken, F. *et al.* Visualizing the effect of an electrostatic gate with angle-resolved photoemission spectroscopy. *Nano Lett.* **19**, 2682–2687 (2019).

6. Jung, J. & MacDonald, A. H. Tight-binding model for graphene π-bands from maximally localized wannier functions. *Phys. Rev. B* **87**, 195450 (2013).

7. Jung, J. & MacDonald, A. H. Accurate tight-binding models for the π bands of bilayer graphene. *Phys. Rev. B* **89**, 035405 (2014).

8. Leconte, N., Javvaji, S., An, J., Samudrala, A. & Jung, J. Relaxation effects in twisted bilayer graphene: A multiscale approach. *Phys. Rev. B* **106**, 115410 (2022).

9. Plimpton, S. Fast parallel algorithms for short-range molecular dynamics. *J. Comput. Phys.* **117**, 1–19 (1995).

10. Thompson, A. P. *et al.* LAMMPS - a flexible simulation tool for particle-based materials modeling at the atomic, meso, and continuum scales. *Comput. Phys. Commun.* **271**, 108171 (2022).

11. Ouyang, W., Mandelli, D., Urbakh, M. & Hod, O. Nanoserpents: Graphene Nanoribbon Motion on Two-Dimensional Hexagonal Materials. *Nano Lett.* **18**, 6009–6016 (2018).

12. Wen, M., Carr, S., Fang, S., Kaxiras, E. & Tadmor, E. B. Dihedral-angle-corrected registry-dependent interlayer potential for multilayer graphene structures. *Phys. Rev. B* **98**, 235404 (2018).

13. Brenner, D. W. *et al.* A second-generation reactive empirical bond order (REBO) potential energy expression for hydrocarbons. *J. Phys. Condens. Matter* **14**, 783–802 (2002).

14. Ku, W., Berlijn, T. & Lee, C.-C. Unfolding first-principles band structures. *Phys. Rev. Lett.* **104**, 216401 (2010).

15. Lee, C.-C., Yamada-Takamura, Y. & Ozaki, T. Unfolding method for first-principles LCAO electronic structure calculations. *J. Phys. Condens. Matter* **25**, 345501 (2013).

16. Nishi, H., Matsushita, Y.-i. & Oshiyama, A. Band-unfolding approach to moiré-induced band-gap opening and Fermi level velocity reduction in twisted bilayer graphene. *Phys. Rev. B* **95**, 085420 (2017).

17. Wanniertools: An open-source software package for novel topological materials. *Comput. Phys. Commun.* **224**, 405–416 (2018).
